# Understanding Machine Learning Applications in Lung Transplantation: A Narrative Review

**DOI:** 10.3389/ti.2025.15640

**Published:** 2026-02-02

**Authors:** Bieke Vercauteren, Balin Özsoy, Jasper Gielen, Meixing Liao, Ewout Muylle, Jan Van Slambrouck, Bart M. Vanaudenaerde, Robin Vos, Pieterjan Kerckhof, Saskia Bos, Jean-Marie Aerts, Laurens J. Ceulemans

**Affiliations:** 1 Department of Chronic Diseases and Metabolism, Laboratory of Respiratory Diseases and Thoracic Surgery (BREATHE), KU Leuven, Leuven, Belgium; 2 Department of Biosystems, M3-BIORES, KU Leuven, Leuven, Belgium; 3 Department of Thoracic Surgery, University Hospitals Leuven, Leuven, Belgium; 4 Department of Oncology, Laboratory of Angiogenesis and Vascular Metabolism, Center for Cancer Biology, VIB, KU Leuven, Leuven, Belgium; 5 Department of Respiratory Diseases, University Hospitals Leuven, Leuven, Belgium

**Keywords:** machine learning, artificial intelligence, transplantation, lung transplantation (LTx), review of literature

## Abstract

Lung transplantation (LTx) offers life-saving therapy for patients with end-stage lung disease but remains limited by donor shortages, complex postoperative management and graft failure. Machine learning (ML) enables opportunities to address these challenges by identifying patterns in complex, high-dimensional data, thereby providing novel insights and improving outcomes. This review outlines ML studies in LTx and explains the methodologies. ML has demonstrated promising results in organ allocation and outcome prediction. Techniques such as support vector machines, and deep learning are useful in risk stratification, while methods like random forests improve interpretability and transfer learning supports model development in data-scarce settings. ML has a growing role in multi-omics data and imaging-based diagnostics. Despite promising results, barriers such as small datasets, cross-center inconsistency, poor interpretability, and limited external validation, hinder clinical adoption. Future progress requires multicenter collaborations, transparent methodologies, and integration within clinical workflows. ML should serve as complementary tool that enhances decision-making, rather than replacing clinical judgement. With careful implementation, it holds the potential to improve transplant outcomes.

## Introduction

Lung transplantation (LTx) is a life-saving treatment for end-stage lung disease. Despite surgical and perioperative advances, challenges remain, including donor shortage, primary graft dysfunction (PGD), and chronic lung allograft dysfunction (CLAD). As clinical data expand and pathophysiology is better understood, these challenges also increase in complexity. Traditional decision-making and predictive modelling is therefore limited.

Machine learning (ML), can identify complex, non-linear patterns, supporting outcome prediction and personalized care [1–5]. In solid organ transplantation, ML is increasingly used to predict survival and improve organ allocation [6]. Nonetheless, integration in LTx lags behind due to small, heterogeneous datasets and complex pathways [7].

The aim of this narrative review is twofold. First, to provide clinicians with a conceptual foundation that fosters understanding of ML. Second, to explore ML applications in LTx, covering outcome prediction, organ allocation, imaging, omics, and other applications.

## Principles of Machine Learning

**FIGURE 1:**
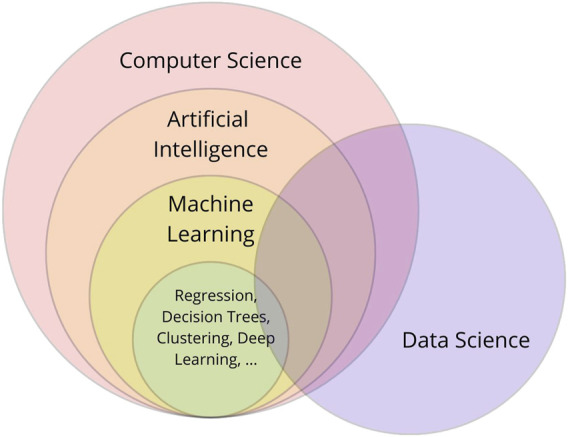
Interrelationship between computer science, artificial intelligence, machine learning, and data science: a conceptual overview.

ML enables mathematical models to learn from data, identify patterns, and make predictions with minimal human intervention. By leveraging algorithms, ML models extract insights and predict outcomes [1]. ML is a central component of artificial intelligence (AI) and closely connected to data science and computer science. These domains overlap (Figure 1) in methodologies, applications, and objectives, making clear distinction difficult [1, 3–5].

ML employs datasets specific for the task. In medical datasets, clinical factors (e.g., age, smoking) serve as *dimensions* (features), while individual observations (e.g., patients, images) represent *samples* (data points). Based on whether labeled data (samples with known outputs) are used, ML approaches can be classified as supervised, unsupervised, and semi-supervised [1–5, 8].

**FIGURE 2:**
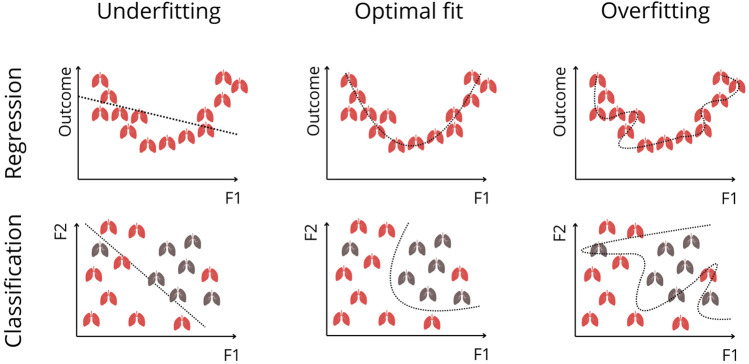
Visualization of Underfitting, Optimal Fitting, and Overfitting in Regression and Classification. The top row illustrates regression settings, where the Outcome axis represents a continuous clinical measure (e.g., survival probability, biomarker level), and F1 represents a predictive feature. Every depicted lung represents a sample (e.g., patient). Underfitting occurs when the model is too simple to capture the true nonlinear relationship, whereas overfitting occurs when the model follows noise instead of the underlying trend. The optimal fit captures the true pattern without modeling random fluctuations. The bottom row shows these concepts in classification, where F1 and F2 represent two predictive features, and each lung corresponds to an individual patient belonging to one of two outcome classes (e.g., favorable vs. poor outcome). The model’s decision boundary is shown as a dotted line. A linear boundary underfits when classes are not linearly separable. An overly complex boundary overfits by tailoring itself to noise and outliers. The optimal fit provides a smooth, generalizable separation between classes.

Supervised ML uses *labeled data* to train predictive models [1–5, 8]. To ensure generalizability, datasets are divided into *training, validation, and testing subsets*. Models first learn patterns from the *training set*. The *validation set* aids in hyperparameter tuning (e.g., batch size, learning rate). It detects underfitting and overfitting, meaning that the model is too simple to capture the true patterns, or learns the noise in the data, respectively (Figure 2) [1–3, 8]. *Cross-validation* is used to ensure generalizability by partitioning the dataset into training and validation subsets. An approach is *k-fold cross-validation,* which divides data randomly into *k* (a number) folds. The model is trained on *k-1* folds and validated on the remaining one, repeating this process *k* times so each subset serves as validation once [1, 2, 5, 8]. Cross-validation ensures the model outcomes are robust and not dependent on a single random split of the dataset [1, 2, 5, 8]. Finally, the *test set*, an unseen portion of data, is used to evaluate the final model performance [1, 2, 8].

Supervised ML is used for *classification* and *regression*. Both utilize labeled datasets, but differ in output: *classification* predicts categories, *regression* predicts continuous values [1, 2, 5, 8].

**FIGURE 3:**
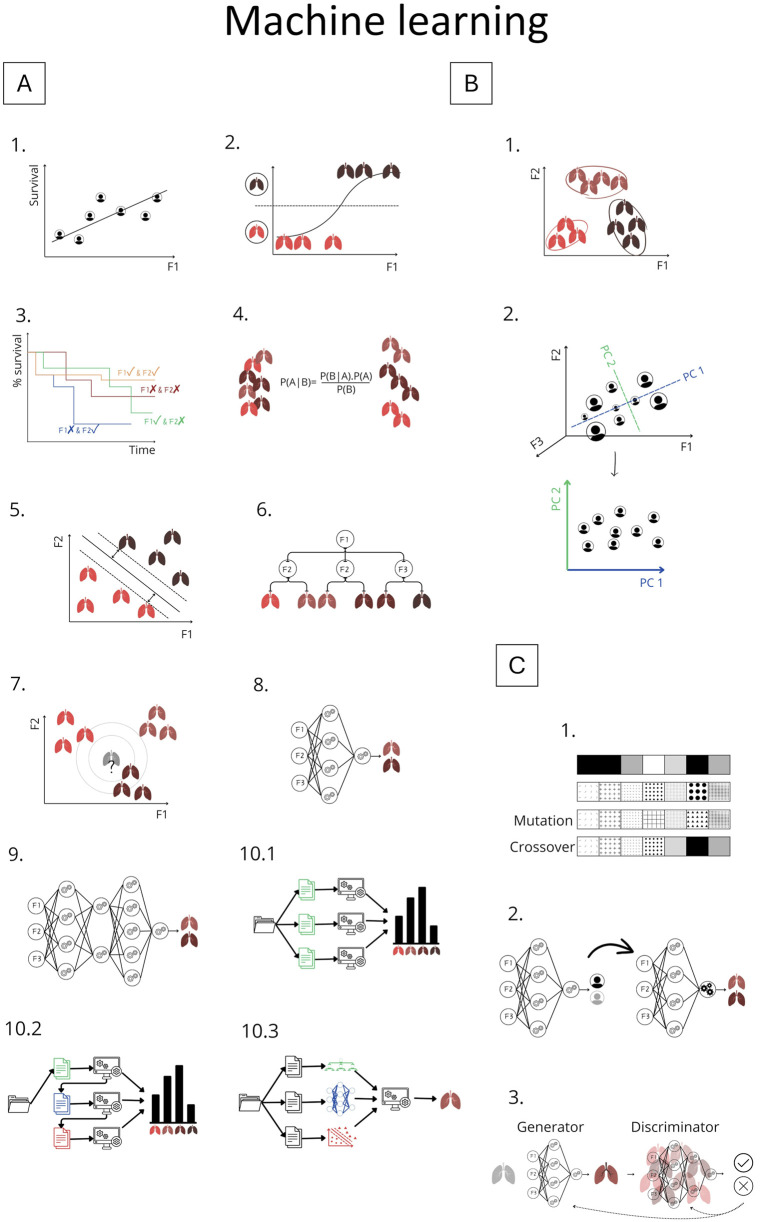
Overview of Machine Learning Methods Explained in Chapter 2. Panel **(A)** Supervised learning methods: **A.1** Linear regression; **A.2** Logistic regression; **A.3** Cox regression; **A.4** Naive Bayes; **A.5** Support vector machine; **A.6** Decision tree; **A.7** k-Nearest Neighbors; **A.8** Artificial neural network; **A.9** Deep learning; **A.10** Ensemble methods: **A.10.1** Bagging, **A.10.2** Boosting, **A.10.3** Stacking. Panel **(B)** Unsupervised learning: **B.1** K-means clustering; **B.2** Principle component analysis. Panel **(C)** Advanced methods: **C.1** Genetic algorithm; **C.2** Transfer learning; **C.3** Generative adversarial network (GAN). F1-F3: represents features; P1-PC2 represents principle components.

Conversely, unsupervised ML analyzes *unlabeled data* to identify patterns [1–3, 5, 8]. Choosing between supervised and unsupervised learning can be difficult, particularly when labeled data are scarce. Semi-supervised ML bridges this gap by combining limited labeled data alongside many unlabeled samples, useful in medical research where data annotation is resource-intensive [1, 2, 8]. Commonly used ML methods, shown in Figure 3, are evaluated and compared using diverse metrics (Table 1).

**TABLE 1:** Common metrics used in machine learning.

Number	Metric	ML type	Description	Common use case
1	Accuracy	Classification	Proportion of correct predictions among total samples	General performance for balanced binary/multiclass classification
2	Mean squared error (MSE)	Regression	Average of squared differences between predicted and true values	Penalize large errors
3	Root mean squared error (RMSE)	Regression	Square root of MSE	Interpretability with penalties
4	Precision	Classification	Proportion of true positives among predicted positives	When false positives are costly (e.g., spam filter)
5	Recall/sensitivity	Classification	Proportion of true positives among actual positives	When false negatives are costly (e.g., disease detection)
6	Specificity	Classification	Proportion of true negatives among actual negatives	When false positives must be avoided (e.g., excluding innocent suspects)
7	Area under the receiver operating characteristic curve (AUROC)	Classification	Area under the receiver operating characteristic curve, combination recall and false positive rate (sometimes interchanged with AUC)	Binary classification, model comparison
8	F1-score	Classification	Harmonic mean of precision and recall	Imbalanced classification
9	Confusion matrix	Classification	Table showing true positives, false positives, true negatives and false negatives	Detailed prediction breakdown
10	Gini index	Classification	Measure of impurity used in splits	Decision tree splitting criterion
11	C-statistic (concordance)	Classification	Probability that the model correctly ranks outcomes	Ranking in survival analysis
12	R^2^ score	Regression	Explained variance ratio	Model fit evaluation
13	Silhouette score	Clustering	Cohesion and separation of clusters	Cluster validation
14	Intraclass inertia	Clustering	Compactness of the clusters, average of the distances between the centroids and the datapoints	Cluster validation

## State-of-the-Art of Machine Learning in Lung Transplantation

LTx involves a heterogeneous, limited patient population with extensive data. LTx recipients have worse outcome than other solid organ transplant recipients, highlighting persistent gaps. ML could contribute to personalized treatment and improved outcomes, as seen in other transplants [5, 9, 10].

The following section reviews key studies, as far as we know (2004–2025), organized into: (1) outcome prediction, (2) organ allocation, and (3) imaging, omics, and other applications. A summary is presented in Table 2. Studies using simpler, borderline-ML methods are excluded from the main text but included in Table 2 and Figure 3.

**TABLE 2:** Overview of Studies about machine learning in lung transplantation.

Autor(s) (Year)	Study population	Input	Output	Model(s)	Metrics	Train/Validation/Test and validation method	Transparency and explanations of ML (mathematical background, architecture, …)
**Outcome prediction**							
Troiani and Carlin [11]*	30 LTx recipients (over 60 subject-years)	2-week epochs of daily/biweekly FEV1 and symptom data	Prediction of acute bronchopulmonary disease events	Heuristic rule-based, classical linear-logistic regression, Bayesian models	Bayesian model AUROC = 0.882 Sensitivity = 0.886 Specificity = 0.955	2-fold cross-validation	Detailed model descriptions, Bayesian priors disclosed, transparency limited in heuristic model
Oztekin et al. (2009) [12]	16604 heart-LTx patients (UNOS)	283 features (demographics, health-related and transplant-related)	9-year graft survival	DTs, ANNs, *logistic regression, Cox regression*	MLP Accuracy = 0.859 Sensitivity = 0.847 Specificity = 0.869	10-fold cross-validation	Hazard function, metrics, k-fold cross-validation, no insight in ML models (brief explantation)
Delen et al. [13]	106398 thoracic patients (UNOS)	565 features (demographics, health-related and transplant-related)	Graft survival time, risk groups	SVM, ANN,DTs, Cox regression and k-means, 2-step, heuristic clustering	SVM MSE = 0.023 R^2^ = 0.879 k-means clustering 3 risk groups intraclass intertia = 1,68 × 10^−8^	10-fold cross-validation	Hazard function, metrics, k-fold cross-validation, no insight in ML models (brief explantation)
Oztekin et al. [14]	6512 LTx records (UNOS)	25 features	Predict LTx success (graft survival and quality of life)	Structural equation modeling (meaning: Statistical method showing how different factors are related to each other, including hidden (latent) ones) DT	R^2^ = 0.68	10-fold cross-validation	Mathematical methodology: Structural equation modeling and composite scores, metrics, k-fold cross-validation
Pande et al. [15]	509 LTx patients (9471 FEV1 evaluations over time)	Time-series FEV1, demographic and clinical features	Predict FEV1 over time and key feature-time interactions	Boosted DTs	RMSE = 0.115–0.421	In sample cross-validation	Models, algorithms, cross-validation, metrics
Oztekin et al. [16]	3684 LTx records (UNOS)	147 features	Predict quality of life post LTx	GA-kNN, GA-SVM, and GA-ANN	GA-SVM Accuracy = 0.994 Precision = 0.991–0.997 Sensitivity = 0.992–0.998 Specificity = 0.996–0.998 F1 = 0.991–0.995	5-fold cross-validation	Normalization, GA, k-fold cross-validation, metrics
Mark et al. [17]	LTx candidates: 1010 IRD, 12013 non-IRD and 19217 waitlist (UNOS)	Top 5 (out of >100 features): recipient and donor characteristics, IRD status, time on waitlist (UNOS)	Compare 5-year survival for IRD vs. non-IRD organ offers	Cox Proportional Hazards, random forests (500 DTs)	7.2% 5-year survival with IRD lung vs. non-IRD 69.9% of simulations favored IRD lung RMSE = 5.3	5-fold cross-validation	RF details
Fessler et al. [18]	410 double LTx recipients	284 patient, donor, and surgical variables in 12 stages	Predict one-year post-transplant mortality	RF	AUROC = 0.65–0.75	Train/test (80/20), 40 repetitions	Limited
Braccioni et al. [19]	24 bilateral LTx recipients	24 recipients variables, incremental cardio-pulmonary exercise testing	Associations between the severity of symptoms (dyspnea, muscle effort, muscle pain) and exercise testing parameters	RF/Boruta	-	5-fold cross-validation (10 resamples)	Limited but short explanation RF/Boruta
Fessler et al. [20]	478 double LTx recipients	6 recipient, donor, intraoperative features in 9 stages	Predict PGD3	Gradient boosting algorithm, SHAP	AUROC = 0.7–0.87	Train/test (80/20)	Limited
Amini et al. [21]	9864 adult US LTx recipients	171 features (demogragics, clincal, transplant)	Classify short-term (≤1 year) vs. long-term (≥10 years) survival after LTx	RF, DT, gradient boosted trees, kNN, ANN, SVM, logistic regression, SHAP	RF Accuracy = 0.7792 Sensitivity = 0.7626 Specificity = 0.7958 AUROC = 0.79	10-fold cross-validation	SHAP
Tian et al. (2023) [22]	504 adult LTx recipients	16 out of 22 clinical variables: recipient, donor, surgical and post-op factors	Predict overall survival	RF, Cox regression	RF integrated AUROC = 0.879 (better than Cox: Integrated AUROC = 0.658)	Train/test split (70/30), bootstrapping (1000 resamples)	Variable importance, overal limited
Melnyk et al. [23]*	369 patients, 125 cases	11 significant out of all preoperative recipient characterstics, procedural variables, perioperative blood product transfusions, and donor charactersitics	Relation between blood transfusion and morbitity (6 endpoints)	Elastic Net regression	Accuracy = 0.765 Sensitivity: 0.80 Specificity: 0.69	Cross-validation (500 repeats)	Limited
Tian et al. [24]	381 LTx patients	15 features: recipient and postoperative	Prediction of airway stenosis requiring clinical intervention	56 models: 7 features selection methods combined with 8 ML models	RF + determination coefficient AUROC = 0.760 Sensitivity = 0.782 Specificity = 0.689	Bootstrap validation (1000 resamples)	Limited
Moro et al. [25]	27296 LTx recipients (UNOS)	60 recipient and donor data	1-, 5-, 10-year survival propabilities	DT; stepwise logistic regression for variable selection	Logisitic regression Accuracy = 0.653 8 subgroups (DT)	Train/test split (70/30), 10-fold cross-validation	Logistic model, DT given, training explantation limited
Michelson et al. [26]	576 bilateral LTx recipients (UNOS, Unet, local)	11 out of 100 donor, recipient pretransplant features	Prediction of PGD3 within 72 h after LTx	LASSO + kNN, logistic regression, XGBoost, SVM, SHAP	kNN AUROC = 0.65 F1 = 0.62	Train/test split (75/25), 5-fold cross-validation (training set 50 resamples)	TRIPOD, preprocessing but limited info about ML, model hosted at pgdcalc.wustl.edu
Xia et al. [27]	802 LTx recipients	9 out of 37 features: Clinical	Predict PGD3 within 72 h post-transplant	9 models (DT, kNN, MLP,RF, SVM, …), SHAP, LASSO	RF: Internal validation AUROC = 0.7975 Sensitivity = 0.7520 Specificity = 0.7313	Train/validate/test split (56/24/20), 5-fold cross-validation	Limited, but visualizations and some information about RF
Fessler et a. [28]	477 LTx patients	66 features in 9 stages	Predict PGD3 at 72h	XGBoost, logistic regression, SHAP	XGBoost: AUROC = 0.84 Sensitivity = 0.81 Specificity = 0.68	Train/test split (80/20) (500 resamples), grid search approach, 5-fold cross-validation	XGBoost model hyperparameter tuning
**Organ allocation**							
Dueñas-Jurado et al. [29]	404 LTx cases	36 donor-recipient variables (clinical, surgical, functional)	Predict 6-month graft survival; optimize donor-recipient matching	Linear regression initial covariates and product units neural networks (LRIPU) model	-	Train/test1/test2 (70/13/17)	Model and coefficients
Zafar et al. [30]	15124 double LTx recipients (UNOS)	19 out of 42 recipient, donor, and transplant variables	Predict 1-, 5-, 10-year survival and half-life; and classify into risk clusters	Cox-LASSO, backward Cox and RF-Cox, clustering via expectation-maximization (LAPT)	Cox-LASSO C statistic for 1-year survival = 0.67 C statistic for 5-year survival = 0.64 C statistic for 10-year survival = 0.72	Train/test (70/30)	Limited
Brahmbhatt et al. [31]	19900 adult LTx patients (UNOS)	Pre-transplant recipient data	Prediction of 1- and 3-year post-transplant mortality	LAS, Houston Methodist model, clinician model, LASSO, RF	RF AUROC = 0.62 Specificity = 0.76 Sensitivity = 0.44 (similar to all other models)	Train/test split (85/15)	Limited
Sage et al. [32]	725 EVLP donor lung assessments	Recipient, donor and 24 EVLP variables	Predict transplant suitability/extubation <72h	XGBoost (InsighTx model), RF	AUROC: 0.75–0.85	Train/test (80/20), 5-fold cross-validation	Code shared
Pu et al. [33]	4610 subjects	Demographics and computed tomography scans	Prediction of left/right/total lung volume, thoracic cavity volume, and heart volume to improve size matching	CNN, 8 ML models (Incl. RF, kNN, DTs)	MLP right and left lung, thoracic cavity R^2^ = 0.501–0.628 XGBoost heart and total lungs R^2^ = 0.430–0.514	Train/validate/test (80/10/10), 10-fold cross-validation	10-Fold cross-validation, visualisations, hyperparameters
Dalton et al. [34]	13204 LTx candidates and 20763 recipients (SRTR)	Demographics and clinical features	Prediction of waitlist mortality at 1, 3, 6 months and post-transplant survival at 1, 3, and 5 years	Cox regression (LAS/lung Composite allocation score), re-estimated models, RF, linear discriminant analysis, logistic regression, boosted DT	Waitlist AUROC = 0.85–0.93 Transplant survival AUROC = 0.56–0.62	10-fold cross-validation	Model explanation in the authors’ Supplementary Material
**Imaging, omics and other applications**							
Bartholmai et al. [35]	119 subjects with interstitial lung disease	High-resolution computed tomography, pulmonary function tests, clinical data	Quantitative classification of interstitial lung disease patterns (emphysema, ground glass, honeycombing, normal and reticular) with correlation to physiology and clinical outcomes	Computer aided lung Informatics for pathology evaluation and rating (CALIPER), ANN, Bayes, SVM, kNN	Analysis of similarity within a cluster R = 0.962	-	Limited, feature extraction
Barbosa et al. (2017) [36]	176 LTx patients	Quantitative Computed tomography scans, PFT, semi-quantitative Computed tomography scores	Diagnose BOS	Multivariate logistic regression, SVM, PCA	Quantitative Computed tomography SVM PCA AUROC = 0.817	10-fold cross-validation (90%–10%)	Limited
Weigt et al. [37]	17 LTx recipients, 1 year post-LTx BAL samples	BAL cell pellet transcriptome (microarray); 40 genes with differential expression (immune-related)	Prediction of incipient CLAD within 2 years post-BAL	Unsupervised hierarchial clustering, SVM, PCA	SVM Accuracy = 0.941	Leave-one-out cross-validation	Limited
Barbosa et al. [38]	71 LTx recipients	Quantitative Computed tomography scans, PFT	Predict eventual onset of BOS	SVM	Accuracy = 85% (3 features); sensitivity = 73.3%; specificity = 92.3%	Train/test (80/20 or 90/10) with 500 or 100 random combinations	Limited
Halloran et al. [39]	242 single-piece LTx biopsies (transbronchial biopsies)	Gene expression (microarrays), 453 rejection-associated transcripts	Identify disease states/phenotypes: normal, T cell mediated rejection, antibody mediated rejection, injury	Unsupervised archetypal analysis, PCA	-	-	Limited, sum of scores
Cantu et al. [40]	113 LTx patients	Clinical, recipient, donor and transplant features, preprocurement donor lung biopsies (gene expression of innate immunity: Toll-like receptor and nod-like receptor pathways)	Prediction of PGD3 at 48–72h post-transplant	Feed-forward deep learning	Toll-like receptor AUROC = 0.776 Sensitivity = 0.786 Specificity = 0.706	5-fold cross-validation	Architecture DL model
Halloran et al. [41]	243 mucosal biopsies from 214 LTx patients	Gene expression (microarrays), 315 rejection-associated transcripts (RATs), 11 pathogenesis based transcripts	Classification into molecular phenotypes: normal, rejection, late inflammation, injury	Unsupervised archetypal analysis, PCA	-	-	Limited, metrics in the authors’ Supplementary Material
Halloran et al. [42]	457 transbronchiale and 314 mucosale biopsies	Gene expression (microarray), rejection-associated transcripts	Prediction of graft survival based on molecular T cell mediated rejection phenotype	Unsupervised archetypal analysis, PCA, RF	-	-	Limited, metrics in the authors’ Supplementary Material
Dugger et al. [43]	49 LTx recipients (small airway brushes and transbronchial biopsies)	RNAseq and digital RNA counts	Diagnosis of CLAD and prediction of graft survival	LASSO logistic regression, RF	RF airway brushing AUROC = 0.84 Transbronchial biopsies AUROC = 0.62	Leave-one-out cross-validation	Limited
Berra et al. [44]	40 LTx patients (BAL)	Protein expession (incl. Angiotensin II-related)	CLAD development	Linear discriminant analysis, SVM, Bayes, quadratic discriminant analysis	CLAD vs. no-CLAD AUROC = 0.86 CLAD development AUROC = 0.97	Leave-one-out cross-validation	Limited
McInnis et al. [45]	88 CLAD patients post-LTx	Computed tomography scans	CLAD phenotype prediction and graft survival prognosis based on lung texture (ML and radiologist scores): Normal, hyperlucent, reticular, ground-glass, honeycomb	Computer-aided lung Informatics for pathology evaluation and rating, Cox regression	Sensitivity: 0.90 Specificity: 0.71 Accuracy: 0.75 AUROC: 0.851	-	Limited
Tran-Dinh et al. [46]	40 LTx recipients	Plasma levels of soluble CD31, oxygenation ratio and respiratory sequential organ failure assement score at 24h/48h/72h	Predict acute cellular rejection within 1 year after LTx	Deep convolutional neural network using time series of biomarkers and multivariate modeling	AUROC = 0.85 Accuracy = 0.87 precision = 0.93 Recall = 0.33–1 (depending on class)	Stratified k-fold cross-validation and external test set with class weighting	Network architecture, modeling methods, time series handling and statistical background
Zhang et al. [47]	243 LTx patients (mucosal biopsies)	Gene expression profiles (19420 genes)	Prediction of 4 clinical response subtypes post-LTx: no rejection, rejection, late inflammation–atrophy, recent injury	Feature selection: boruta and others Classifiers: SVM, RF, kNN, DT	SVM Accuracy = 0.992 (247 genes used)	10-fold cross-validation	Metrics
Su et al. [48]	59 LTx recipients, 181 sputum samples	16S rRNA microbiota sequencing and clinical biomarkers (procalcitonin, T-lymphocyte levels)	Differentiate infection vs. acute rejection vs. event-free	RF, linear discriminant analysis	Infection vs. event-free AUROC = 0.898 Rejection vs. event-free AUROC = 0.919 Infection vs. rejection AUROC = 0.895	10-fold crossvalidation	Limited
Watzenboeck et al. [49]*	19 LTx recipients (BAL)	Microbiome (16S rRNA), metabolome, lipidome, BAL cell composition, clinical data, lung function tests	Predict FEV1 changes at 30/60/90 days (lung function trajectory)	ridge regression models	30 days r = 0.76 60 days r = 0.63 90 days r = 0.42	Nested cross-validation (train: 3-fold cross-validation, test: 4-fold cross-validation)	Limited
Stefanuto et al. [50]	35 LTx recipients, 58 BAL and blind bronchial aspirate samples	VOC profiles (386 features, reduced to 20 features)	Predict severe (PGD3) vs. mild/no PGD (PGD0–2)	SVM	AUROC = 0.90 Accuracy = 0.83 Sensitivity: 0.63 Specificity: 0.94	Train/test (50/50), leave-one-out cross-validation	Limited, visualisation of ML pipline
Qin et al. [51]	97 human LTx paired biopsies (pre/post-LTx)	Expression profiles (microarrays, incl. transcriptomics for cuproptosis-related genes)	Diagnosis of lung ischemia–reperfusion injury, identification of cuproptosis-related biomarkers	LASSO, SVM + recursive feature elimination, RF, logistic regression	15 biomarker, for each AUROC >0.8 Logisitic regression AUROC = 0.96	Train/test (53/47), validation in rat model	Limited
Wijbenga et al. [52]*	152 LTx recipients	Exhaled breath via SpiroNose (7-sensor eNose); patient and clinical characteristics	Diagnosis of CLAD and discrimination of phenotypes	Partial least squares discriminant analysis, logistic regression	AUROC = 0.94 Specificity = 0.78 Sensitivity = 1 Discrimination BOS vs. Restrictief allograft syndroom AUROC = 0.95	Train/test (67:33); 10-fold cross-validation	Limited
Ram et al. [53]*	80 out of 100 donor lung pairs (Computed tomography-imaged *ex situ*)	*Ex vivo* CT scans, donors and recipient features	Donor lung suitability classification; prediction of ICU stay, PGD3 and 2-year CLAD	Dictionary learning (supervised ML) seen as a simpler technique	Accuracy = 0.727 AUROC = 0.743 F-score = 0.75 Precision = 0.78 Recall = 0.74	Train/test split (18/82)	In their Supplementary Material: explanation and formulas dictionary learning, sparse coding, classification, training
Chao et al. [54]	113 donor lungs evaluated with *ex vivo* lung perfusion	Chest radiographs, functional EVLP data	Predict transplant suitability and early post-transplant ventilation outcomes	Extreme gradient boosting (XGBoost)	Combined model AUROC = 0.807 Sensitivity = 0.76 Specificity = 0.89–0.94	75%–25% training-test split, repeated with 30 random seeds	Limited
Gouiaa et al. (2024) [55]	40 LTx patients	Plasma levels of soluble CD31, oxygenation ratio and respiratory sequential organ failure assement score at 24h/48h/72h	Predict acute cellular rejection within 1 year after LTx	Taelcore (topological autoencoder, ANN classifier) compared to other models (incl. RF, kNN)	MSE = 0.307 RMSE = 0.0.38	Stratified k-fold cross-validation; training/test split 75/25%	Topological loss function, persistence homology, entropy, rips filtration, metrics, short explanation other models, open-source code (GitHub)
Gao et al. [56]	113 + 97 lung graft biopsy samples	38 signature genes	Prediction of ischemia–reperfusion injury and PGD	Weighted gene coexpression network analysis, LASSO, RF and nomogram	AUROC >0.70 for all 4 genes	LASSO: 10-fold cross-validation	Limited, small explanations of models
Chen et al. [57]*	160 LTx patients	Demographics, LTx data and 69 lab indicators	Predict time to first rejection	LASSO regression, multivariate Cox model	1 year AUROC = 0.799 2 years AUROC = 0.757 3 years AUROC = 0.892	Train/test (70/30)10-fold cross-validation	Limited
Choshi et al. [58]	117 + 6 LTx patients (87112 datapoints)	36 clinical factors, time series data of tacrolimus doses and route of administration	Predict tacrolimus trough levels	Multivariate long short-term memory: an improved RNN, SHAP	R^2^ = 0.67 Tacrolimus trough levels within ±30% of actual = 88.5%	Train/validate/test (80/10/10)	Metrics

Partitioned in “outcome prediction,” “organ allocation” and “Imaging, omics and other applications,” in chronological order. If an article was not discussed in the text, an asterisk is placed next to it. If multiple models were tested, metrics were reported for best-performing ML methods. ANN, Artificial Neural Network; AUROC, Area Under the Receiver Operating Characteristic Curve; BAL, Bronchoalveolar Lavage; BOS, Bronchiolitis Obliterans Syndrome; CLAD, Chronic Lung Allograft Dysfunction; DL, Deep Learning; DT, Decision Tree; EVLP, *Ex Vivo* Lung Perfusion; FEV1, Forced Expiratory Volume in one second; GA, Genetic Algorithm; IRD, Increased Risk for Disease Transmission; kNN, k-Nearest Neighbors; LAPT, Lung Transplantation Advanced Prediction Tool; LAS, Lung Allocation Score; LASSO, Least Absolute Shrinkage and Selection Operator; LTx, Lung Transplantation; ML, Machine Learning; MLP, Multilayer Perceptron; MSE, Mean Squared Error; PCA, Principal Component Analysis; PFT, Pulmonary Function Test; PGD, Primary Graft Dysfunction; RF, Random Forest; RMSE, Root Mean Squared Error; RNN, Recurrent Neural Network; SHAP, SHapley Additive Explanation; SVM, Support Vector Machine; UNOS, the United Network for Organ Sharing; VOC, Volatile Organic Compound.

### Outcome Prediction

#### Survival and Quality of Life

**FIGURE 4:**
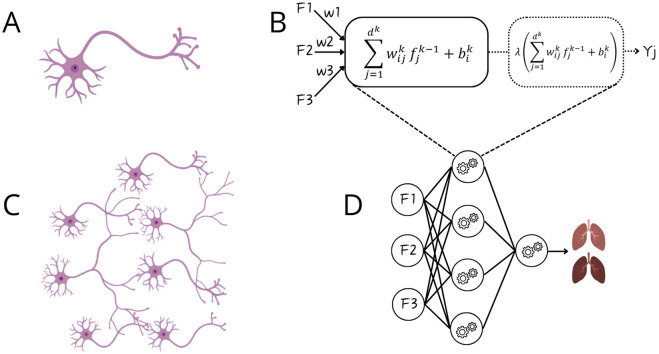
Comparison between biological neurons and artificial neural networks. Panel **(A)** Biological neuron receiving input via dendrites and sending output via axon; Panel **(B)** Artificial neuron, a perceptron, receiving input from features (F1-F3) and after mathematical manipulation sending output as Y_j_ (binary output); Panel **(C)** Connection of multiple neurons via synapses; Panel **(D)** Artificial neural network.

In a series of studies, Oztekin, Delen, Amini and colleagues demonstrated the value of ML for outcome prediction. Initially, they showed that ML outperformed expert–selected variables and traditional statistical models in predicting 9-year graft survival after heart–lung transplantation, identifying more relevant variables and relationships [12]. They applied logistic regression (Supplementary Text, Figure 3A.2), decision trees (DTs), and artificial neural networks (ANNs)*. DTs* (Figure 3A.6) are interpretable models that recursively split data to form rule-based trees. They are sensitive to noise and require pruning (removing unnecessary parts) to improve generalizability [1, 2, 4, 5, 8]. *ANNs* (Figure 3A.8) are algorithms inspired by the brain (Figures 4A–D). The simplest form, a single-layer perceptron, mimics a biological neuron. Adding hidden layers, referring to synaptic connections creates a multilayer perceptron (MLP) [1, 2, 4, 5, 8]. Unlike DTs, ANNs lack interpretability and rely on large datasets, therefore, the United Network for Organ Sharing (UNOS) cohort of 16,604 patients was crucial for this approach [5, 8].

**FIGURE 5:**
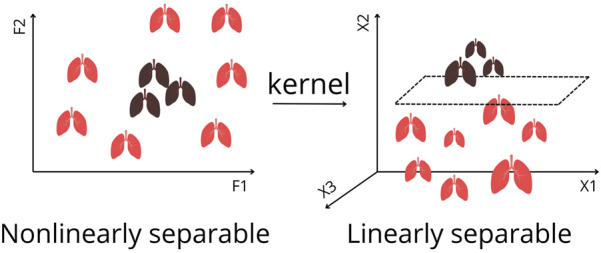
Kernel Trick for Nonlinearly Separable Data in Feature Space to Linearly Separable Data. By applying a kernel function, the data are transformed into a higher dimension, where a separating hyperplane can be found. This enables Support Vector Machines to classify complex patterns that cannot be separated in the original feature space. F1, F2 represents features. X1, X2, X3 represents axis of projections in a higher dimension.

Later, their work was extended to survival estimation, again comparing ML with expert-selected and literature-based variables. ML outperformed both approaches by retaining important predictors overlooked in traditional methods. They applied DTs and ANNs, and additionally introduced support vector machines (SVMs) [13]. *SVMs* (Figure 3A.5) are algorithms that maximize the margin between classes (distance between the decision boundary and the nearest data points from each class). An innovation is the kernel trick, which enables SVMs to classify nonlinearly separable data by mapping it into higher-dimensional space (Figure 5) [1–5, 8]. Model performance was compared using Cox regression (Supplementary Text, Figure 3A.3). Subsequently, k-means clustering, two-step cluster analysis, and conventional heuristic approaches were used to determine the optimal number of patient risk groups. *Unsupervised k-means clusterin*g (Figure 3B.1) groups data into a predefined number of clusters based on feature similarity by iteratively assigning samples to the nearest centroid (center of a cluster) and updating centroids as the mean of assigned samples. It offers an unbiased way to explore risk groups [1–5, 8]. In this study, three clusters were optimal [13].

In 2011, a DT–based hybrid model was designed to provide an interpretable ML approach. However, its accuracy remained low. Moreover, using variables predefined from previous studies biased the model, potentially missing important interactions [14]. To predict quality of life, Genetic Algorithm (GA)-based approaches for feature selection were introduced [16], particularly useful for complex, feature-rich domains with limited samples as in LTx. *GAs* (Figure 3C.1) are optimization techniques inspired by biological evolution, using selection, crossover, and mutation to find optimal solutions, e.g., determining representative variables [5, 59]. The GA was combined with three classification algorithms: SVM, ANN and k-Nearest Neighbors (kNN) (Figure 3A.7). Unlike other algorithms, *kNN* predicts without training, by averaging outcomes of the k most similar samples to unseen input. Performance depends on data quality, choice of distance metric, and k. In high-dimensional data, kNN’s accuracy can degrade [1, 2, 5, 8], therefore, combining it with GA is appropriate.

Subsequent research performed classification of post-LTx survival (≤1 year vs. ≥10 years), incorporating additional methods, namely ensemble models such as random forests (RF) and gradient boosting trees [21]. *Ensemble learning* combines multiple models to improve predictive accuracy, reduce overfitting, and enhance robustness [1, 2, 5, 8]. *Bagging* (bootstrap aggregating) (Figure 3A.10.1) improves stability by training on different data subsets [1, 2, 5, 8]. *RF* is a common bagging method that aggregates DTs [1, 5, 8]. *Boosting* (Figure 3A.10.2) builds models sequentially, each correcting errors of the previous one [1, 5, 8]. Among all models, RF achieved the best performance. To improve model transparency, the authors employed an explainable AI (XAI) method: *SHapley Additive Explanations* (SHAP), a model-agnostic framework that quantifies each feature’s contribution to a prediction by considering all possible feature combinations [60]. SHAP identified Hepatitis B surface antibody and forced expiratory volume in one second (FEV1) as predictors of long-term survival. However, methodological limitations warrant consideration. The use of binary classification (≤1 year vs. ≥10 years) excluded nearly half of the cohort [21]. This neglects intermediate survival, arguably the most challenging to predict, which makes the modest performance noticeable.

Moro et al. created a DT for survival predictions. Using UNOS data, 47 features were identified via stepwise logistic regression, assuming linear relationships. Consequently, meaningful nonlinear interactions may have been missed, and reducing 60 to 47 variables offered minimal dimensional or computational benefit. The final DT used six key predictors, including three postoperative variables, limiting the model’s preoperative prognostic utility, despite its interpretability. Eight subgroups (decision nodes) showed distinct survival curves. As expected, best outcomes occurred in younger recipients with short hospital stays, limited ventilation support, and no reintubation [25].

To compare survival between increased risk for disease transmission (IRD) organ recipients versus non-IRD organ recipients, Mark et al. applied RF and Cox regression. As Cox regression performed best, it was selected for further analysis, which somewhat diminished the novelty of ML implementation. Nevertheless, the study offered a data-driven perspective to expand the donor pool, demonstrating a 7.2% improvement in 5-year survival for IRD lung transplant recipients [17].

Unlike the prior study, Tian et al. demonstrated that RF can outperform Cox regression, for survival prediction under standard conditions, achieving high predictive accuracy. Generalizability across subgroups with different diagnoses and treatments was reported. However, the single-center design and limited sample size may question this [22].

The effectiveness of RF, combined DTs, was also shown by Fessler et al., analyzing 284 variables across 12 perioperative stages to predict one-year mortality. As presumed, the accuracy went up by including information of later stages. Lung allocation score (LAS) emerged as top predictor [18].

#### Primary Graft Dysfunction

A subsequent study by Fessler et al. used gradient boosting to predict PGD3, a syndrome linked to adverse outcomes [61]. Extracorporeal membrane oxygenation use, along with recipient factors, were revealed as top predictors [20]. Due to the short length of these papers [18, 20], the information provided on the ML implementation is limited. In their most recent paper [28], predicting PGD3 at 72h, they offer more information about logistic regression and *XGBoost*, an efficient gradient boosting variant, that improves computational memory usage, well-suited for large datasets [62]. Fessler’s studies introduce an innovative approach by progressively incorporating data from successive transplant phases, allowing the prognosis to be refined at each stage.

Michelson et al. similarly predicted PGD3 using pretransplant data, enabling potential application in patient selection and pretransplant counseling. From 100 features, Least Absolute Shrinkage and Selection Operator (LASSO) (Supplementary Text) selected 11 predictors. Among four models, kNN performed best and was released as open-access risk calculator [26].

With data from 802 patients, Xia et al. evaluated nine algorithms. RF classified PGD3 best. SHAP identified blood loss as important, but prior feature selection, based on linear relation assumption, may have introduced selection bias [27].

#### Other Outcome Parameters

Using a small, unbalanced dataset, Tian et al. developed eight ML models combined with seven feature selection methods to predict airway stenosis requiring clinical intervention. Key predictors in RF included postoperative 6-minute walk test and indication for LTx. This model could guide postoperative follow-up [24].

Braccioni et al. assessed how clinical parameters relate to symptom severity during exercise testing after LTx. *Boruta*, a feature selection method based on RF [63], revealed associations for limited exercise capacity: dyspnea correlating with peak ventilation and work rate, muscle effort with breathing reserve, and muscle pain with VO_2_ peaks. These findings linked reduced aerobic capacity and high ventilatory cost to symptom severity. DT visualizations offered interpretable insights to guide exercise prescriptions [19]. Despite the small dataset (n = 24), the authors justified using ML, noting the method performs well in small, high-dimensional datasets without assuming normality or independence. Nonetheless, small cohorts increase overfitting risk and limit generalizability of the findings.

To analyze repeated FEV1 measurements after LTx, Pande et al. developed a longitudinal model, handling challenges as within-subject correlation, unequal time intervals, and unbalanced designs. Although FEV1 typically declines over time, patterns vary with individual factors. The method was clearly described and implemented in an R package [15].

Overall, the studies reviewed above show the potential of ML in LTx, but the applications stay rather limited. Stronger tools, e.g., deep learning (DL), could be implemented, as seen in section *Organ Allocation* [33].

### Organ Allocation

LTx faces suboptimal organ allocation, causing long wait times and significant candidate mortality [64]. Varying donor selection criteria across centers limits organ availability. Allocation studies suffer from bias, as unaccepted organs are absent in training datasets. Unlike other transplants with comprehensive donor-recipient risk stratification, LTx allocation largely neglects the combined influence of factors [30].

To address these challenges, Zafar et al. developed the LTx Advanced Prediction Tool (LAPT). Based on 15,124 UNOS cases, LAPT grouped patients into low-, medium-, and high-risk subsets. LAPT outperformed LAS by predicting 1-, 5-, and 10-year survival and graft half-life for donor-recipient matches. This web-based tool enables data-driven allocation beyond recipient-centric systems [30].

Dueñas-Jurado et al. combined logistic regression with ANNs for donor-recipient matching. They incorporated donor, recipient and perioperative variables to predict 6-month graft survival, claiming to outperform traditional methods, although metrics were not reported. Key predictors included low pre-transplant CO_2_, while prolonged donor ventilation, older donor and recipient age were linked to poorer outcomes [29].

To assess the suitability of donor lungs, Sage et al. created InsighTx, a RF model integrating *ex vivo* lung perfusion (EVLP) and other variables, offering a quantitative approach to evaluate and improve lung utilization [32]. However, its primary endpoint, extubation time, serves only as a short-term proxy for success and does not fully capture longer-term outcomes.

Pu et al. developed eight ML models using donor demographics to predict lung, heart, and thoracic cavity volumes, to improve donor-recipient size matching [33]. The performance of these approaches was benchmarked against convolutional neural network (CNN)-based image segmentation models, which were used to generate the volumetric ground truth. *CNNs* are a class of *DL* (Figure 3A.9), referring to ANNs with multiple hidden layers, designed to process structured grid-like data like images. They use filters to detect local structures (e.g., edges) and combine them to recognize shapes. Like other DL models, it requires large labeled datasets and significant processing power [1–3, 8]. The best-performing model was a MLP for individual lungs and thoracic cavity estimates. These non-imaging-based volume predictions may enhance allocation [33].

In contrast to these optimistic findings, Brahmbhatt et al. concluded that LAS, clinician-based models, LASSO, and RF are not sufficiently accurate to predict post-LTx survival. LAS overestimated mortality in high-risk patients and the AUROC of the Houston Methodist model was not achieved, highlighting challenges of reproducibility and possible overfitting in earlier literature. Predictive performance was not improved by ML, disease-specific models, or donor variables [31].

Similarly, Dalton et al. reported that LAS refinement and advanced techniques did not improve performance. Seven models were evaluated with waitlist and post-transplant data to predict waitlist mortality or post-transplant survival. While waitlist models showed strong discrimination, all post-transplant models performed poorly [34]. A possible solution is integrating images or biological markers. Studies employing these approaches are examined in section *Imaging, Omics and Other Applications*.

### Imaging, Omics and Other Applications

Barbosa et al. investigated quantitative CT (qCT) to diagnose bronchiolitis obliterans syndrome (BOS), a form of CLAD. Logistic regression and SVM were used to compare qCT metrics, pulmonary function tests (PFT), and semi-quantitative imaging scores as input. To reduce qCT dimensionality, principal component analysis (PCA) (Figure 3B.2) was applied, projecting the data onto components capturing the highest variance while minimizing information loss [1, 2, 5, 8]. PCA of qCT together with PFT outperformed all models. However, BOS diagnosis relied solely on chart-reviewed PFT decline, creating circularity, lacking pathological confirmation, and potentially biasing comparisons between qCT- and PFT-based models [36]. In a subsequent study, qCT features including lobar volumes, airway volumes, and airway resistance differed significantly in BOS patients, even at baseline. Using SVM, they constructed classifiers in one-, two-, and three-dimensional feature spaces. Remarkably, with only three qCT parameters, the model achieved 85% accuracy in predicting BOS [38]. Bartholmai et al. also used qCTs, to develop the CALIPER platform for interstitial lung diseases. They applied different ML methods to categorize lung parenchyma into five patterns, challenging even for expert readers to distinguish. CALIPER provided 3D visualizations for tracking of disease burden [35]. Later, McInnis et al. tested CALIPER to distinguish CLAD phenotypes and predict graft survival. Both CALIPER and radiologist scores independently predicted graft failure, with CALIPER enabling reproducible phenotyping and early prognostication without requiring expiratory CT [45]. An XGBoost model based on X-rays and perfusion data from EVLP was developed to predict transplant suitability and ventilation duration post-LTx. Abnormalities were scored per lobe and correlated with oxygenation, compliance and edema. SHAP ranked consolidation and infiltrates as strongest associated with function and transplantability [54]. These studies illustrate how ML-driven imaging analysis can overcome interobserver variability, provide objective and reproducible quantification, reduce human workload, and enable more accurate, scalable assessment of graft injury.

Tran-Dinh et al. developed a model to predict acute cellular rejection using soluble CD31 (sCD31) as biomarker. From only forty recipients, sCD31 levels were combined with recipient haematosis in a CNN model [46]. The authors claim their model uses concepts similar to *transfer learning* (Figure 3C.2), where a model trained on one task is adapted to another, valuable in data-scarce settings [1, 2]. However, this is questionable, as their network was trained from scratch rather than optimized from a pretrained model. In another study, a topological autoencoder (Taelcore) was created to improve these predictions by capturing underlying data structures. Applied to the same dataset, dimensionality reduction with Taelcore achieved more accurate predictions than methods like PCA [55]. Likewise, features extracted by Taelcore lack biological interpretability.

To predict tacrolimus trough levels (TTLs) in LTx patients, Choshi et al. developed a long short-term memory–based *Recurrent Neural Network* (RNN), a DL model handling sequential data. This approach relied on clinical inputs identified by SHAP, including previous TTLs and tacrolimus doses. The model captured temporal patterns in dosing and drug response, enabling individualized immunosuppressant management [58]. Yet, its accuracy may diminish in real-world patient settings where missed doses and irregular timing are common.

A gene expression–based DL classifier by Cantu et al. used preprocurement donor lung biopsies to predict PGD3. Their Toll-like receptor model outperformed clinical covariates [40], demonstrating strong discriminative ability and indicating donor innate immune activation as a key driver of PGD, though the analysis was limited to two pathways. Gao et al. also used transcriptomic data in different algorithms. Four neutrophil extracellular traps-related hub genes were identified as drivers of ischemia-reperfusion injury. Three of these were validated in clinical samples, related with PGD development [56]. Furthermore, transcriptomic data were used to explore cuproptosis, a form of cell death, as a potential mechanism in ischemia-reperfusion injury. Three methods (LASSO, SVM, RF) recognized critical biomarkers, with good performance. Functional enrichment linked these genes to immune regulation and cell death, while immune infiltration analysis revealed associations with distinct immune cell subsets [51].

Using unsupervised ML on LTx transbronchial biopsies, Halloran et al. defined four rejection archetypes. PCA linked T-cell mediated rejection (TCMR) and injury to T cell and macrophage transcripts, and antibody-mediated rejection-like to endothelial markers [39]. They also showed that this method worked for mucosal biopsies [41]. However, because mucosal biopsies were obtained only during protocol or clinically indicated bronchoscopies, the sampling may be biased toward unwell patients, limiting generalizability to asymptomatic recipients. Molecular TCMR was associated with future graft loss. Molecular scores outperformed clinical variables in RF and remained robust even in low-surfactant or mucosal samples [42]. Across these studies, Halloran et al. demonstrate that molecular profiling of biopsies provides a more biologically coherent assessment of rejection than histology, although the work remains limited by sampling bias, nonspecific injury signals, and small sample size. Using previously reported mucosal biopsy data [41], Zhang et al. classified recipients into four rejection-related subgroups. Supervised classification achieved high accuracies (likely overfitted: more features than samples) and lacked external validation. Predictive genes were linked to T cell signaling and innate immunity [47].

In another study, lymphocytic bronchitis gene signature in transbronchial biopsies and small airway brushings were used to predict graft failure and differentiate CLAD from controls. Gene expression profiling with RF showed superior diagnostic performance for brushings over biopsies, but because brushings contain mixed epithelial and leukocyte populations, cell-type–specific interpretation remains limited. The lymphocytic bronchitis score was elevated in CLAD and associated with 2.4-fold increased risk of graft loss [43].

Su et al. analyzed 181 sputum samples from 59 recipients using 16S rRNA sequencing, classifying samples into “stable”, “infection”, and “rejection”. Differences in microbial composition appeared, with six genera enriched during acute rejection, suggesting immune-modulatory roles. Integrating these genera and clinical data in a RF classified well, though repeated samples per patient may cause biased results [48]. A study by Weigt described that gene expression profiling of cells in bronchoalveolar lavage (BAL) revealed an immune activation signature preceding clinical CLAD diagnosis. Forty genes were differentially expressed in incipient CLAD versus CLAD-free samples, enriched for cytotoxic lymphocyte markers. SVM achieved 94.1% accuracy in distinguishing only seventeen cases [37]. Berra et al. also used BAL samples to predict CLAD and investigate the association with the renin–angiotensin system. Although single proteins could not discriminate, combinations in ML classifiers can, reflecting ML’s strength in modelling beyond human assessment [44].

Another study predicted PGD using volatile organic compounds (VOCs) from BAL fluid and bronchial aspirate samples. VOC profiling with SVM modeling achieved 83% accuracy in distinguishing PGD3 from lower grades. Twenty VOCs, associated with lipid peroxidation and oxidative stress, were top predictors. Additional analyses linked VOC patterns to clinical variables, including donor BMI and Organ Care System, indicating potential confounding. Recipient and intraoperative factors did not significantly influence VOC profiles [50].

## Key Insights, Future Directions and Conclusion

A consistent strength of ML is its ability to integrate many weak or noisy features into a meaningful signal, where human interpretation or single-variable analyses fail. ML can capture complex, nonlinear interactions, reveal hidden patterns, and offer early risk stratification that traditional clinical or statistical methods miss. Yet, the limitations across studies are strikingly uniform. Most studies are small, single-center, only internally validated, and based on imbalanced datasets. Sampling bias, missing confounders, and heterogeneous data quality further reduce generalizability. Compared with kidney, liver, and heart transplantation, where ML-based tools are more mature, ML approaches in LTx research remains largely underexplored [65–74]. Reporting is often insufficient: many papers provide limited mathematical detail about model design, preprocessing, hyperparameter tuning, or validation, making replication difficult and hindering fair comparison across studies. More transparent, standardized reporting following frameworks like MI-CLAIM (Minimum Information about Clinical Artificial Intelligence Modeling) and TRIPOD-AI (Transparent Reporting of a Multivariable prediction model for individual Prognosis Or Diagnosis) should be strongly encouraged.

### Future Directions & Underused Advanced Methods

Future directions should include more multimodal datasets, true external validation, and the careful use of advanced ML methods. Stacking, an ensemble model, could improve performance by combining diverse base learners and a meta learner. Generative Adversarial Networks (GANs) could augment datasets. They consist of a generator that creates synthetic data and a discriminator that evaluates authenticity. Through adversarial training, based on unlabeled data, both networks iteratively improve, allowing to generate realistic data. Although the information content does not increase, it enhances model flexibility and generalization. These are only two examples of underused ML methods, that could strengthen model performance. Post-hoc explanation tools such as Local Interpretable Model-agnostic Explanations [5] and SHAP will remain essential to ensure that predictions are clinically interpretable.

### Conclusion

ML holds major potential in LTx, from improving outcome prediction and organ allocation, to imaging and omics-based insights. Yet, clinical adoption remains limited due to small, single-center datasets and insufficient external validation. Enhancing generalizability and building trust requires large multicenter studies, XAI, and standardized reporting. Additionally, ethical considerations remain important when using ML in medicine [2]. Progress in other solid organ transplants highlights opportunities for LTx, with techniques still unexplored, offering room for future innovation. Crucially, ML should complement clinical decision-making, and not replace clinical judgement. Its success relies on collaboration among clinicians, data scientists, ethicists, and regulators. Overcoming current barriers will enable ML to meaningfully improve transplant outcomes.

## Glossary

AI: Artificial Intelligence
ANN: Artificial Neural Network
AUROC: Area Under the Receiver Operating Characteristic Curve
Bagging: Bootstrap Aggregating
BAL: Bronchoalveolar Lavage
BMI: Body Mass Index
BOS: Bronchiolitis Obliterans Syndrome
CLAD: Chronic Lung Allograft Dysfunction
CNN: Convolutional Neural Network
DL: Deep Learning
DT: Decision Tree
EVLP: *Ex Vivo* Lung Perfusion
FEV1: Forced Expiratory Volume in one second
GA: Genetic Algorithm
GAN: Generative Adversarial Network
IRD: Increased Risk for Disease Transmission
kNN: k-Nearest Neighbors
LAPT: Lung Transplantation Advanced Prediction Tool
LAS: Lung Allocation Score
LASSO: Least Absolute Shrinkage and Selection Operator
LTx: Lung Transplantation
ML: Machine Learning
MLP: Multilayer Perceptron
MSE: Mean Squared Error
PCA: Principal Component Analysis
PFT: Pulmonary Function Test
PGD: Primary Graft Dysfunction
qCT: quantitative Computed Tomography
RF: Random Forest
RMSE: Root Mean Squared Error
RNN: Recurrent Neural Network
sCD31: soluble CD31
SHAP: SHapley Additive Explanation
SVM: Support Vector Machine
TCMR: T-cell-mediated Rejection
TTLs: Tacrolimus Trough Levels
UNOS: the United Network for Organ Sharing
**VO**_**2**_: Volume of Oxygen Consumption
VOC: Volatile Organic Compound
XAI: explainable artificial intelligence
