# Supplementary material for: Understanding Machine Learning Applications in Lung Transplantation: A Narrative Review
Source: Transpl Int. 2026 Feb 2;38:15640. doi: 10.3389/ti.2025.15640 (PMC12908661; doi:10.3389/ti.2025.15640)
Supplement: Supplementary file 1 [file Supplementaryfile1.docx]

## **Supervised learning techniques**

### Linear regression

Linear regression (**Figure 3A.1**), used for regression tasks, models the relationship between a dependent variable (outcome, a numerical value, $y$) and one or more independent variables (predictors or features, $f$; number of predictors, $n$). Starting from initial guesses for each weight ($w$), it iteratively adjusts them to minimize the difference between predicted values ($\hat{y}$) and actual outcomes ($y$): $\hat{y}=w_{0}+w_{1}f_{1}+w_{2}f_{2}+\ldots+w_{n}f_{n}$. Linear methods has difficulties with capturing non-linear relationships and is sensitive to outliers^1,4,8^.

### Logistic regression

Logistic regression (**Figure 3A.2**) is a supervised algorithm used for binary classification, predicting the probability of each class using a sigmoid function:
$P\left( Y=1 | F \right)=\frac{1}{1+e^{-(w_{0}+w_{1}f_{1}+w_{2}f_{2}+\ldots+w_{n}f_{n})}}$. It is rapidly trained and well-suited for data-limited fields like transplantation^1^. However, obstacles are again outliers and non-linear relationships^1,3,7^.

### Cox regression

Cox regression (**Figure 3A.3**) predicts time-to-event outcomes like transplant failure or death, while accounting for multiple risk factors. It relies on the proportional hazards assumption, which states that each variable’s effect remains constant over time^9^.

To improve risk factor selection and prevent overfitting, *Least Absolute Shrinkage and Selection Operator (LASSO)* can be utilized. LASSO applies a penalty that shrinks less relevant coefficients, making it useful for large datasets^10^.

### Naive Bayes

Bayes (**Figure 3A.4**) is a simple yet effective classifier based on Bayes' theorem, which updates outcome probability using prior knowledge and new evidence: $P\left( A | B \right)= \frac{P(B|A)\cdot P(A)}{P(B)}$ . P(A∣B) is the updated probability of A given B, P(B∣A) is the likelihood of B given A, P(A) is the prior probability of A, and P(B) is the overall probability of B. Bayesian models handle uncertainty and improve accuracy as more patient information becomes available^1,2,4,11^. A simplified form, Naive Bayes, assumes that all features are independent. Although this assumption is rarely valid in real-world scenarios, this model often performs well. However, its accuracy decreases with strong feature dependencies^5,7^.
